# Supplementary material for: Enhanced DNA Repair Pathway is Associated with Cell Proliferation and Worse Survival in Hepatocellular Carcinoma (HCC)
Source: Cancers (Basel). 2021 Jan 17;13(2):323. doi: 10.3390/cancers13020323 (PMC7830462; doi:10.3390/cancers13020323)
Supplement: Supplementary file 1 [file cancers-13-00323-s001.pdf]

Supplemental Materials: Enhanced DNA Repair Pathway is Associated with Cell Proliferation and Worse Survival in Hepatocellular Carcinoma (HCC)

Masanori Oshi, Tae Hee Kim, Yoshihisa Tokumaru, Li Yan, Ryusei Matsuyama, Itaru Endo, Leonid Cherkassky and Kazuaki Takabe

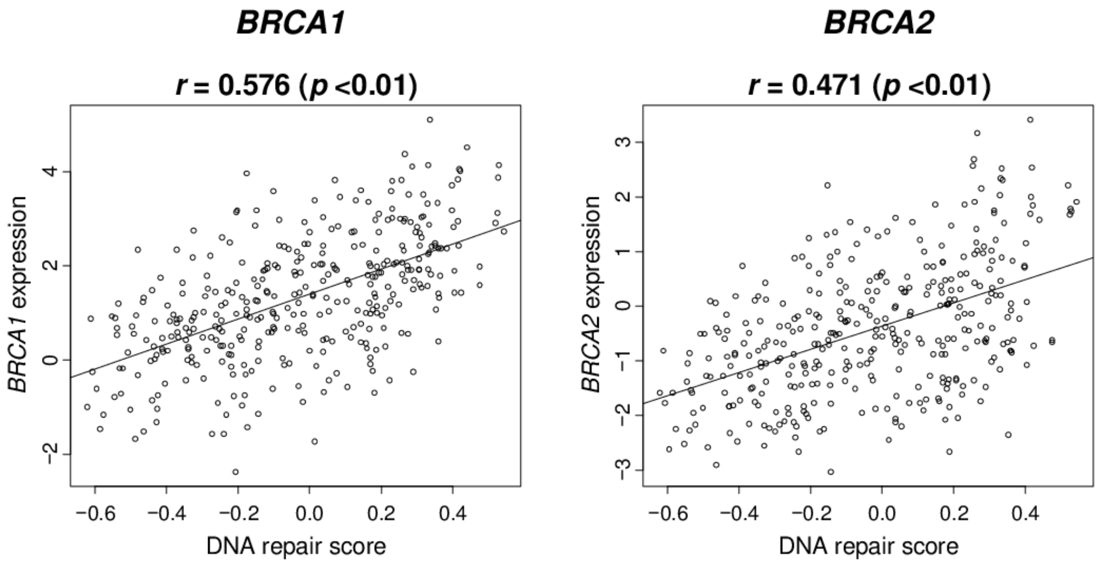

Figure S1. The correlation of the DNA repair score with expression of *BRCA1* and *BRCA2* genes in the TCGA cohort. Correlation plots of the DNA repair score with expression of the *BRCA1* and *BRCA2* genes. Spearman's rank correlation coefficient was used to calculate the *p*-value.

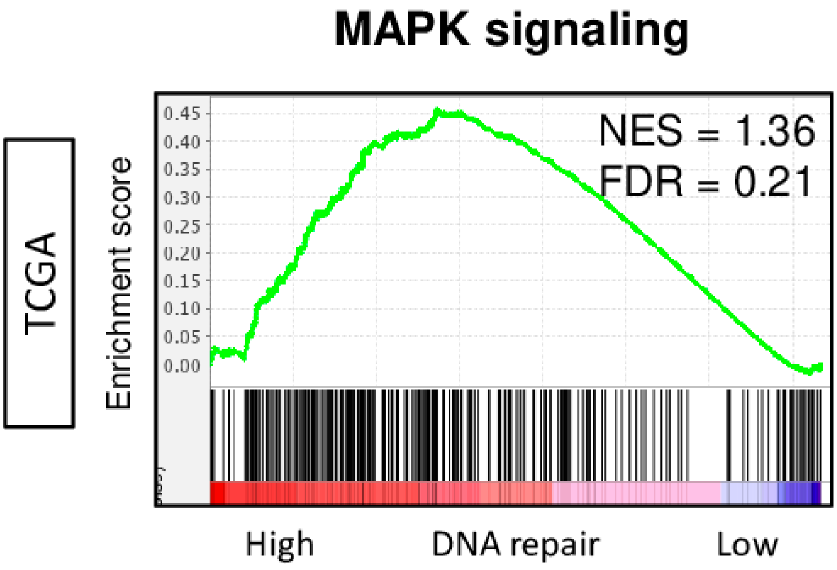

Figure S2. Gene set enrichment analysis (GSEA) of the KEGG MAPK signaling gene sets by high vs low DNA repair score of HCC in the TCGA cohort. Enrichment plots along with normalized enrichment score (NES) and false discovery rate (FDR) for the KEGG MAPK signaling gene set. An FDR of 0.25 was used to deem statistical Scheme 3. The correlation of the DNA repair score with drug sensitivity of Lenvatinib, Cabozantinib, Sorafenib, and Regorafenib in HCC cell lines. The correlation plots are between the DNA repair score and the level of drug sensitivity area under curve (AUC) of Lenvatinib, Cabozantinib, Sorafenib, and Regorafenib in HCC cell lines. Gene expression data of the HCC cell lines and response to drugs were obtained from the DepMap

portal, which were HEP3B217, HLF, HUH1, HUH7, JHH2, JHH4, JHH5, JHH6, JHH7, PLCPRF5, SKHEP1, SNU182, SNU387, SNU398, SNU423, SNU449, SNU475, SNU761, SNU878, and SNU886. Spearman correlation statistics were used for the analysis.

Table S1. Genes included in the Molecular Signatures Database (MSigDB) Hallmark DNA repair gene set.

Gene Symbol	Gene description
**AAAS**	aladin WD repeat nucleoporin
**ADA**	adenosine deaminase
**ADCY6**	adenylate cyclase 6
**ADRM1**	adhesion regulating molecule 1
**AK1**	adenylate kinase 1
**AK3**	adenylate kinase 3
**APRT**	adenine phosphoribosyltransferase
**ARL6IP1**	ADP ribosylation factor like GTPase 6 interacting protein 1
**BCAM**	basal cell adhesion molecule
**BCAP31**	B cell receptor associated protein 31
**BOLA2**	bolA family member 2
**BRF2**	BRF2 RNA polymerase III transcription initiation factor subunit
**MPC2**	mitochondrial pyruvate carrier 2
**CANT1**	calcium activated nucleotidase 1
**CCNO**	cyclin O
**CDA**	cytidine deaminase
**CETN2**	centrin 2
**CLP1**	cleavage factor polyribonucleotide kinase subunit 1
**CMPK2**	cytidine/uridine monophosphate kinase 2
**NELFB**	negative elongation factor complex member B
**COX17**	cytochrome c oxidase copper chaperone COX17
**CSTF3**	cleavage stimulation factor subunit 3
**DAD1**	defender against cell death 1
**DCTN4**	dynactin subunit 4
**DDB1**	damage specific DNA binding protein 1
**DDB2**	damage specific DNA binding protein 2
**GSDME**	gasdermin E
**DGCR8**	DGCR8 microprocessor complex subunit
**DGUOK**	deoxyguanosine kinase
**DUT**	deoxyuridine triphosphatase
**EDF1**	endothelial differentiation related factor 1
**EIF1B**	eukaryotic translation initiation factor 1B
**AGO4**	argonaute RISC component 4
**ELL**	elongation factor for RNA polymerase II
**ERCC1**	ERCC excision repair 1, endonuclease non-catalytic subunit
**ERCC2**	ERCC excision repair 2, TFIIH core complex helicase subunit
**ERCC3**	ERCC excision repair 3, TFIIH core complex helicase subunit
**ERCC4**	ERCC excision repair 4, endonuclease catalytic subunit
**ERCC5**	ERCC excision repair 5, endonuclease
**ERCC8**	ERCC excision repair 8, CSA ubiquitin ligase complex subunit
**FEN1**	flap structure-specific endonuclease 1
**GMPR2**	guanosine monophosphate reductase 2
**GPX4**	glutathione peroxidase 4
**GTF2A2**	general transcription factor IIF subunit 2
**GTF2B**	general transcription factor IIB
**GTF2F1**	general transcription factor IIF subunit 1
**GTF2H1**	general transcription factor IIH subunit 1
**GTF2H3**	general transcription factor IIH subunit 3
**GTF2H5**	general transcription factor IIH subunit 5
**GTF3C5**	general transcription factor IIIC subunit 5
**GUK1**	guanylate kinase 1

**HCLS1**	hematopoietic cell-specific Lyn substrate 1
**HPRT1**	hypoxanthine phosphoribosyltransferase 1
**IMPDH2**	inosine monophosphate dehydrogenase 2
**ITPA**	inosine triphosphatase
**LIG1**	DNA ligase 1
**MPG**	N-methylpurine DNA glycosylase
**MRPL40**	mitochondrial ribosomal protein L40
**NCBP2**	nuclear cap binding protein subunit 2
**NFX1**	nuclear transcription factor, X-box binding 1
**NME1**	NME/NM23 nucleoside diphosphate kinase 1
**NME3**	NME/NM23 nucleoside diphosphate kinase 3
**NME4**	NME/NM23 nucleoside diphosphate kinase 4
**NPR2**	natriuretic peptide receptor 2
**NT5C**	5′, 3′-nucleotidase, cytosolic
**NT5C3A**	5′-nucleotidase, cytosolic IIIA
**NUDT21**	nudix hydrolase 21
**NUDT9**	nudix hydrolase 9
**PCNA**	proliferating cell nuclear antigen
**PDE4B**	phosphodiesterase 4B
**PDE6G**	phosphodiesterase 6G
**PNP**	purine nucleoside phosphorylase
**POLA1**	DNA polymerase alpha 1, catalytic subunit
**POLA2**	DNA polymerase alpha 2, accessory subunit
**POLB**	DNA polymerase beta
**POLD1**	DNA polymerase delta 1, catalytic subunit
**POLD3**	DNA polymerase delta 3, accessory subunit
**POLD4**	DNA polymerase delta 4, accessory subunit
**POLE4**	DNA polymerase epsilon 4, accessory subunit
**POLH**	DNA polymerase eta
**POLL**	DNA polymerase lambda
**POLR1C**	RNA polymerase I and III subunit C
**POLR1D**	RNA polymerase I and III subunit D
**POLR2A**	RNA polymerase II subunit A
**POLR2C**	RNA polymerase II subunit C
**POLR2D**	RNA polymerase II subunit D
**POLR2E**	RNA polymerase II subunit E
**POLR2F**	RNA polymerase II subunit F
**POLR2G**	RNA polymerase II subunit G
**POLR2H**	RNA polymerase II subunit H
**POLR2I**	RNA polymerase II subunit I
**POLR2J**	RNA polymerase II subunit J
**POLR2K**	RNA polymerase II subunit K
**POLR3C**	RNA polymerase III subunit C
**POLR3GL**	RNA polymerase III subunit G like
**POM121**	POM121 transmembrane nucleoporin
**PRIM1**	DNA primase subunit 1
**RAD51**	RAD51 recombinase
**RAD52**	RAD52 homolog, DNA repair protein
**RAE1**	ribonucleic acid export 1
**RALA**	RAS like proto-oncogene A
**RBX1**	ring-box 1
**NELFE**	negative elongation factor complex member E
**REV3L**	REV3 like, DNA directed polymerase zeta catalytic subunit
**RFC2**	replication factor C subunit 2
**RFC3**	replication factor C subunit 3
**RFC4**	replication factor C subunit 4
**RFC5**	replication factor C subunit 5
**RNMT**	RNA guanine-7 methyltransferase
**RPA2**	replication protein A2

**RPA3**	replication protein A3
**RRM2B**	ribonucleotide reductase regulatory TP53 inducible subunit M2B
**SAC3D1**	SAC3 domain containing 1
**SDCBP**	syndecan binding protein
**SEC61A1**	SEC61 translocon subunit alpha 1
**SF3A3**	splicing factor 3a subunit 3
**SMAD5**	SMAD family member 5
**SNAPC4**	small nuclear RNA activating complex polypeptide 4
**SNAPC5**	small nuclear RNA activating complex polypeptide 5
**SRSF6**	small nuclear RNA activating complex polypeptide 6
**SSRP1**	structure specific recognition protein 1
**STX3**	syntaxin 3
**SUPT4H1**	SPT4 homolog, DSIF elongation factor subunit
**SUPT5H**	SPT5 homolog, DSIF elongation factor subunit
**SURF1**	SURF1 cytochrome c oxidase assembly factor
**TAF10**	TATA-box binding protein associated factor 10
**TAF12**	TATA-box binding protein associated factor 12
**TAF13**	TATA-box binding protein associated factor 13
**TAF1C**	TATA-box binding protein associated factor, RNA polymerase I subunit C
**TAF6**	TATA-box binding protein associated factor 6
**TAF9**	TATA-box binding protein associated factor 9
**TARBP2**	TARBP2 subunit of RISC loading complex
**ELOA**	elongin A
**NELFCD**	negative elongation factor complex member C/D
**ALYREF**	Aly/REF export factor
**TK2**	thymidine kinase 2
**TMED2**	transmembrane p24 trafficking protein 2
**TP53**	tumor protein p53
**TSG101**	tumor susceptibility 101
**TYMS**	thymidylate synthetase
**UMPS**	uridine monophosphate synthetase
**UPF3B**	UPF3B regulator of nonsense mediated mRNA decay
**USP11**	ubiquitin specific peptidase 11
**VPS28**	VPS28 subunit of ESCRT-I
**VPS37B**	VPS37B subunit of ESCRT-I
**VPS37D**	VPS37D subunit of ESCRT-I
**XPC**	XPC complex subunit, DNA damage recognition and repair factor
**ZNF707**	zinc finger protein 707
**ZNRD1**	zinc ribbon domain containing 1
**ZWINT**	ZW10 interacting kinetochore protein

Table S2. Association between disease-specific survival and expression of multiple genes in the DNA repair pathway in the TCGA cohort. The Cox proportional hazards regression model with disease-specific survival was used in the analysis.

Gene	HR	95% CI			p
**NME1**	1.45	1.23	-	1.70	0.0000
**SAC3D1**	1.56	1.29	-	1.90	0.0000
**NELFCD**	1.61	1.28	-	2.02	0.0001
**TAF9**	1.52	1.24	-	1.88	0.0001
**POLR2I**	1.49	1.22	-	1.82	0.0001
**ZWINT**	1.26	1.12	-	1.41	0.0001
**POLD1**	1.36	1.15	-	1.59	0.0002
**NT5C3A**	1.41	1.17	-	1.69	0.0003
**RAE1**	1.50	1.20	-	1.86	0.0003
**EDF1**	1.44	1.18	-	1.76	0.0004
**NT5C**	1.38	1.15	-	1.65	0.0004
**RFC5**	1.37	1.15	-	1.64	0.0005
**LIG1**	1.34	1.13	-	1.58	0.0006

**POLA1**	1.30	1.12 -	1.52	0.0007
**RPA3**	1.43	1.16 -	1.76	0.0008
**POLR1C**	1.44	1.16 -	1.78	0.0008
**RAD51**	1.22	1.09 -	1.38	0.0009
**DAD1**	1.51	1.19 -	1.93	0.0009
**RALA**	1.46	1.16 -	1.82	0.0010
**AAAS**	1.53	1.18 -	1.96	0.0011
**PCNA**	1.32	1.12 -	1.57	0.0011
**SUPT4H1**	1.44	1.16 -	1.80	0.0012
**TAF12**	1.45	1.16 -	1.81	0.0012
**UPF3B**	1.35	1.12 -	1.62	0.0014
**RFC4**	1.28	1.10 -	1.49	0.0017
**PRIM1**	1.25	1.09 -	1.43	0.0019
**GTF3C5**	1.45	1.15 -	1.84	0.0019
**FEN1**	1.26	1.09 -	1.46	0.0020
**TAF10**	1.47	1.15 -	1.89	0.0020
**ERCC3**	1.39	1.13 -	1.72	0.0022
**SSRP1**	1.38	1.12 -	1.70	0.0024
**RFC2**	1.33	1.11 -	1.60	0.0025
**DCTN4**	1.41	1.13 -	1.77	0.0028
**SUPT5H**	1.42	1.13 -	1.78	0.0029
**POLA2**	1.31	1.10 -	1.57	0.0031
**SF3A3**	1.40	1.12 -	1.76	0.0035
**NELFE**	1.28	1.09 -	1.52	0.0035
**SNAPC4**	1.36	1.11 -	1.67	0.0035
**ALYREF**	1.28	1.08 -	1.51	0.0039
**POLE4**	1.29	1.08 -	1.53	0.0040
**IMPDH2**	1.29	1.08 -	1.54	0.0046
**GTF2H1**	1.38	1.10 -	1.73	0.0061
**POLR2D**	1.41	1.10 -	1.79	0.0062
**ELOA**	1.40	1.10 -	1.78	0.0062
**ITPA**	1.33	1.08 -	1.63	0.0063
**POLR2J**	1.33	1.08 -	1.64	0.0066
**APRT**	1.30	1.08 -	1.57	0.0066
**NME3**	1.30	1.08 -	1.57	0.0067
**POLR3C**	1.32	1.07 -	1.62	0.0081
**POLB**	1.26	1.06 -	1.50	0.0087
**RFC3**	1.21	1.05 -	1.40	0.0094
**POLR2K**	1.33	1.07 -	1.65	0.0103
**TARBP2**	1.33	1.07 -	1.64	0.0103
**ERCC8**	1.34	1.07 -	1.69	0.0110
**POLR2H**	1.30	1.06 -	1.59	0.0118
**AK1**	1.24	1.04 -	1.47	0.0144
**RPA2**	1.30	1.05 -	1.60	0.0146
**POLD3**	1.23	1.04 -	1.45	0.0156
**TAF1C**	1.27	1.04 -	1.54	0.0185
**TYMS**	1.15	1.02 -	1.29	0.0208
**NELFB**	1.31	1.04 -	1.65	0.0224
**GTF2A2**	1.33	1.04 -	1.70	0.0233
**CSTF3**	1.29	1.03 -	1.60	0.0241
**GTF2H3**	1.22	1.02 -	1.46	0.0258
**CANT1**	1.25	1.03 -	1.53	0.0264
**ADCY6**	1.20	1.02 -	1.42	0.0280
**CETN2**	1.25	1.02 -	1.52	0.0290
**ERCC1**	1.26	1.02 -	1.54	0.0300
**TAF13**	1.25	1.02 -	1.52	0.0317
**ZNRD1**	1.22	1.02 -	1.47	0.0333
**NME4**	1.18	1.01 -	1.38	0.0346
**NUDT21**	1.23	1.01 -	1.50	0.0370

**ARL6IP1**	1.21	1.01	-	1.45	0.0370
**POLR3GL**	1.23	1.01	-	1.49	0.0375
**DGUOK**	1.26	1.01	-	1.56	0.0378
**SEC61A1**	1.26	1.01	-	1.57	0.0430
**POLL**	1.29	1.01	-	1.65	0.0435
**POLR2C**	1.22	1.00	-	1.49	0.0447
**TSG101**	1.29	1.00	-	1.65	0.0458
**STX3**	1.15	1.00	-	1.33	0.0507
**RBX1**	1.25	1.00	-	1.56	0.0527
**UMPS**	1.25	1.00	-	1.57	0.0530
**DGCR8**	1.23	0.99	-	1.52	0.0615
**ERCC2**	1.22	0.99	-	1.49	0.0623
**PNP**	1.16	0.99	-	1.35	0.0664
**DDB1**	1.24	0.98	-	1.57	0.0671
**POLR2G**	1.22	0.98	-	1.52	0.0691
**EIF1B**	1.24	0.98	-	1.56	0.0713
**NUDT9**	1.20	0.98	-	1.47	0.0761
**MRPL40**	1.24	0.98	-	1.58	0.0769
**NFX1**	1.20	0.98	-	1.48	0.0787
**GUK1**	1.21	0.98	-	1.50	0.0815
**TK2**	1.21	0.98	-	1.50	0.0831
**SRSF6**	1.23	0.97	-	1.56	0.0839
**BRF2**	1.14	0.98	-	1.33	0.0902
**DUT**	1.16	0.97	-	1.38	0.0941
**VPS28**	1.18	0.97	-	1.42	0.0950
**USP11**	1.17	0.97	-	1.41	0.0956
**TAF6**	1.14	0.98	-	1.33	0.0974
**RNMT**	1.16	0.97	-	1.39	0.0975
**ADRM1**	1.22	0.96	-	1.54	0.1082
**BCAP31**	1.16	0.97	-	1.40	0.1121
**POLR1D**	1.19	0.96	-	1.46	0.1145
**POLR2E**	1.22	0.95	-	1.55	0.1156
**ADA**	1.13	0.97	-	1.31	0.1159
**SMAD5**	1.15	0.97	-	1.36	0.1195
**ERCC5**	1.19	0.95	-	1.48	0.1288
**REV3L**	1.12	0.97	-	1.30	0.1354
**VPS37D**	1.12	0.96	-	1.30	0.1526
**MPC2**	1.16	0.94	-	1.43	0.1553
**POLR2F**	1.23	0.92	-	1.63	0.1591
**ZNF707**	1.15	0.94	-	1.40	0.1662
**GTF2F1**	1.18	0.93	-	1.50	0.1718
**HPRT1**	1.12	0.95	-	1.33	0.1750
**SNAPC5**	1.18	0.93	-	1.48	0.1757
**AK3**	1.12	0.94	-	1.33	0.1955
**RRM2B**	1.13	0.94	-	1.35	0.1969
**SURF1**	1.16	0.93	-	1.44	0.2000
**XPC**	1.13	0.93	-	1.36	0.2092
**TMED2**	1.15	0.92	-	1.42	0.2145
**GMPR2**	1.15	0.92	-	1.44	0.2314
**GTF2B**	1.15	0.91	-	1.44	0.2425
**GTF2H5**	1.14	0.91	-	1.43	0.2643
**HCLS1**	0.92	0.80	-	1.06	0.2712
**RAD52**	1.11	0.92	-	1.34	0.2739
**CLP1**	1.14	0.90	-	1.45	0.2757
**VPS37B**	1.09	0.93	-	1.29	0.2772
**POM121**	1.07	0.94	-	1.22	0.2828
**ELL**	1.12	0.91	-	1.39	0.2859
**POLH**	1.08	0.92	-	1.26	0.3362
**COX17**	1.12	0.87	-	1.44	0.3697

**CDA**	1.04	0.95	-	1.14	0.4271
**POLR2A**	1.07	0.90	-	1.27	0.4525
**GPX4**	1.09	0.87	-	1.37	0.4604
**BCAM**	0.96	0.85	-	1.08	0.4949
**NPR2**	0.97	0.87	-	1.07	0.5532
**GSDME**	0.97	0.88	-	1.07	0.5842
**NCBP2**	1.06	0.85	-	1.33	0.5884
**TP53**	0.96	0.82	-	1.12	0.6096
**DDB2**	0.96	0.81	-	1.15	0.6707
**CMPK2**	1.03	0.91	-	1.16	0.6764
**MPG**	1.04	0.85	-	1.27	0.6964
**ERCC4**	1.03	0.88	-	1.20	0.7402
**SDCBP**	1.02	0.86	-	1.21	0.7935
**AGO4**	1.02	0.85	-	1.23	0.8148
**POLD4**	1.00	0.82	-	1.22	0.9801
**PDE4B**	1.00	0.89	-	1.12	0.9812
**CCNO**	1.00	0.94	-	1.07	0.9927

*CI, confidence interval; HR, hazard ratio.
